# Neutrophil-activating secretome characterizes palbociclib-induced senescence of breast cancer cells

**DOI:** 10.1007/s00262-024-03695-5

**Published:** 2024-05-02

**Authors:** Gabriele Favaretto, Marianna Nicoletta Rossi, Lorenzo Cuollo, Mattia Laffranchi, Manuela Cervelli, Alessandra Soriani, Silvano Sozzani, Angela Santoni, Fabrizio Antonangeli

**Affiliations:** 1 Institute of Molecular Biology and Pathology (IBPM), National Research Council (CNR), c/o Department of Molecular Medicine, Sapienza University of Rome, Viale Regina Elena 291, 00161 Rome, Italy; 2 Department of Science, Roma Tre University, Rome, Italy; 3 Department of Molecular Medicine, Sapienza University of Rome, Rome, Italy; 4 Istituto Pasteur Italia-Fondazione Cenci Bolognetti, Rome, Italy; 5 IRCCS Neuromed, Pozzilli, Italy; 6 Present Address: Department of Pediatrics, Columbia University, New York, NY USA

**Keywords:** Breast cancer, Palbociclib, Senescence, SASP, Serum amyloid A, Neutrophil, NET

## Abstract

Senescent cells have a profound impact on the surrounding microenvironment through the secretion of numerous bioactive molecules and inflammatory factors. The induction of therapy-induced senescence by anticancer drugs is known, but how senescent tumor cells influence the tumor immune landscape, particularly neutrophil activity, is still unclear. In this study, we investigate the induction of cellular senescence in breast cancer cells and the subsequent immunomodulatory effects on neutrophils using the CDK4/6 inhibitor palbociclib, which is approved for the treatment of breast cancer and is under intense investigation for additional malignancies. Our research demonstrates that palbociclib induces a reversible form of senescence endowed with an inflammatory secretome capable of recruiting and activating neutrophils, in part through the action of interleukin-8 and acute-phase serum amyloid A1. The activation of neutrophils is accompanied by the release of neutrophil extracellular trap and the phagocytic removal of senescent tumor cells. These findings may be relevant for the success of cancer therapy as neutrophils, and neutrophil-driven inflammation can differently affect tumor progression. Our results reveal that neutrophils, as already demonstrated for macrophages and natural killer cells, can be recruited and engaged by senescent tumor cells to participate in their clearance. Understanding the interplay between senescent cells and neutrophils may lead to innovative strategies to cope with chronic or tumor-associated inflammation.

**Supplementary Information:**

The online version contains supplementary material available at 10.1007/s00262-024-03695-5.

## Introduction

Cellular senescence represents a cellular stress response primarily triggered by DNA damage, including telomere shortening and cytosolic nucleic acid sensing, and it is considered an alternative fate to regulated cell death [1–3]. Senescent cells are characterized by a long-lasting cell cycle arrest and therefore are regarded as a hurdle against tumorigenesis [4]. Nevertheless, senescent cells are metabolically active and many of their biological functions are driven by the so-called senescence-associated secretory phenotype (SASP). This is a complex and temporally regulated program that involves the secretion of bioactive molecules and inflammatory factors in the surrounding microenvironment [5–7]. SASP makes senescent cells crucial in orchestrating immune cell recruitment and tissue plasticity around neoplastic lesions, showing even opposite effects on tumor progression [8]. For instance, senescence-dependent recruitment of lymphocytes of the innate immunity, namely natural killer (NK) cells, can mediate tumor regression [9–11]. NK cells recognize senescent cells expressing the ligands of the activating receptors NKG2D and DNAM-1 and eliminate them through the release of perforin- and granzyme-containing granules [12–16]. Macrophages also participate in the clearance of senescent cells [17, 18]. Furthermore, senescent cells have been recently described to alert the adaptive arm of the immune system by enhancing tumor cell immunogenicity through the priming of dendritic cells and the activation of tumor antigen-specific CD8 T cells [19, 20]. On the other hand, senescence can also establish an immunosuppressive microenvironment [21] and promote cancer cell stemness [22]. In certain contexts, senescent cells may express the inhibitory molecule HLA-E, dampening NK cell, and CD8 T cell effector functions [23], and expand the myeloid-derived suppressor cell (MDSC) compartment within the tumor niche [24]. Much less is known about the interaction between senescent cells and neutrophils, which are the first immune cells recruited to injured tissues. Neutrophils have been reported to target senescent cells during vascular remodeling in retinopathy [25] and to be influenced by the secretome of senescent hepatoma cells with contrasting results on neutrophil extracellular trap (NET) formation capacity [26]. Considering the increasing evidence of a prominent role of neutrophils in the tumor immune landscape, there is an urgent need to investigate the cross talk between senescent cells and neutrophils. According to the state of the art, tumor-associated neutrophils (TANs) can establish antitumor responses by direct killing of cancer cells via reactive oxygen species (ROS) production or by serving as antigen-presenting cells. Conversely, TANs can skew the immune responses toward a tumor-promoting inflammation and a permissive environment driving angiogenesis and extracellular matrix remodeling [27].

Immunotherapy has revolutionized cancer treatment and the therapeutic efficacy of many anticancer drugs relies on their immunomodulatory effects [28, 29]. Cellular senescence has great potential as an immunomodulatory tool due to its intimate connection with the immune system. Pro-senescence therapy of tumor cells may even be considered as a new type of immunotherapy [30]. Palbociclib (PD0332991), ribociclib (LEE011), and abemaciclib (LY2835219) belong to the third generation of CDK4/6 inhibitors, which have been recently approved in association with hormonal therapy for the treatment of hormone receptor-positive and human epidermal growth factor receptor 2-negative (HR + /HER2-) metastatic breast cancer and have promptly demonstrated additional effects beyond the antiproliferative property [31–33]. Palbociclib (Ibrance, Pfizer Inc.), the first licensed, has shown non-canonical functions among which the induction of a senescence-like phenotype in tumor cells has attracted great attention for its implications in cancer pathology [34–37]. In this regard, palbociclib treatment may represent a form of therapy-induced senescence (TIS). Preclinical evidence extends palbociclib application also to tumors other than breast cancer, including leukemias, melanoma, pancreatic carcinoma, head and neck cancer, and glioblastoma [38–40]. Moreover, palbociclib is being tested in clinical trials in combination with immune checkpoint inhibitors or other agents [41, 42]. Noteworthy, a recent alert from the Food and Drug Administration (FDA) warns about severe pulmonary adverse effects following the administration of CDK4/6 inhibitors, including palbociclib, and a preclinical study has shown that palbociclib treatment leads to neutrophil recruitment to fibrotic lung lesions potentially contributing to pulmonary inflammation [43, 44]. Therefore, shedding light on the way palbociclib-induced senescent cells modulate neutrophil behavior is of great relevance. To this aim, in this work we treated human breast cancer cells with palbociclib to study the induction of cellular senescence and to analyze the resulting SASP. Specifically, we assessed the ability of senescent tumor cells to mediate inflammation by recruiting and activating neutrophils.

## Materials and methods

### Cell culture and treatment

Human breast cancer cell lines MCF7 and MDA-MB-231 were kindly gifted by Rossella Maione (Sapienza University of Rome, Italy). Primary human foreskin fibroblasts (HFFs) were from the American Type Culture Collection, ATCC SCRC-1041™. Cells were routinely screened for mycoplasma contamination with the PCR mycoplasma detection kit from abm (G238). Cells were cultured in a humified incubator at 37°C with 5% CO_2_ in DMEM high glucose supplemented with 10% FBS, 2 mM L-Gln, and 100 U/ml penicillin/streptomycin (Euroclone). Optimal seeding density was established for each cell line to reach 70–80% confluence at the experimental endpoint. Palbociclib (PD0332991) was provided by Pfizer Inc. and used at 2 μM which is a standard concentration in cellular studies [45] and not far from physiologically achievable concentrations in the plasma of patients [46].

### Cell cycle analysis

Cells were harvested and fixed in cold 70% ethanol at least overnight at 4°C. After washing in PBS, cells were incubated with 50 μg/ml propidium iodide containing 40 μg/ml RNAse A for 30 min at room temperature and immediately analyzed by flow cytometry with a CytoFLEX cytometer from Beckman Coulter. Data were elaborated using FlowJo software v.10.7.1 (FlowJo, OR, USA), and cell cycle determined with the Dean-Jett-Fox model after doublets exclusion.

### Apoptosis evaluation

Apoptotic and dead cells were detected using the dead cell apoptosis kit with Annexin V FITC and propidium iodide for flow cytometry from Invitrogen (V13242) according to the manufacturer’s instructions.

### SA-β-Gal assay

Senescence-associated β-galactosidase (SA-β-Gal) activity was assessed by using the senescence β-galactosidase staining kit from cell signaling technology (#9860) according to the manufacturer’s instructions. Senescent cells were identified as blue-stained cells by standard light microscopy. Images were acquired using an EVOS microscope with magnification 200 ×.

### Lamin-B1 detection

Lamin-B1 was detected by immunofluorescence microscopy. Cells were fixed with methanol/acetone at ratio 3/7 and stained overnight at 4°C with anti-Lamin-B1 rabbit mAb (Abcam, ab133741) diluted 1:100 in PBS with 5% BSA, 0.3 M glycine, 0.1% Triton X-100. After washing in PBS, AF594-cojugated goat anti-rabbit IgG secondary antibody (Invitrogen, A-11012) was applied for 1 h at room temperature. Cover slip was mounted using SlowFade™ gold antifade mountant with DAPI (Invitrogen, S36938). Images were acquired by conventional epifluorescence microscopy using an Olympus BX51 microscope equipped with a ProgRes® MF cool monochrome camera (Jenoptik) and processed with I.A.S software ver. 009 (Delta Sistemi) for merging and pseudo-coloring adjustment.

### Conditioned medium collection

At the end of the treatment, culture medium containing FBS was replaced with fresh medium without FBS. Treated and untreated cells were cultured in T-25 flasks with 6 ml of medium and, after 24 h, conditioned media were collected, and cells counted. Supernatants were centrifugated (13,000 rpm for 15 min at 4°C) to remove cell debris and stored unconcentrated in aliquots at − 80°C until the day of use. Media were thawed on ice and used undiluted, 100 μl for ELISA analysis and 0.5 ml in 24-well plate for neutrophil functional assays.

### Luminex multiplex immunoassay

Cytokine levels in cell culture supernatants were measured using a custom human premixed multi-analyte kit (R&D Systems) and a Bio-Plex® MAGPIX™ multiplex reader (Bio-Rad Laboratories) according to the manufacturer’s instructions. Levels of CCL2, CCL27, CCL3, CCL4, CCL7, Chemerin, CX_3_CL1, CXCL10, CXCL9, GM-CSF, IFN-γ, IL-10, IL-12, IL-13, IL-15, IL-18, IL-1α, IL-1β, IL-2, IL-21, IL-28A, IL-4, IL-5 were measured. Samples were run in duplicate and cytokine concentrations were calculated using a six-point standard curve derived from measurement of serially diluted panel-specific standards. Upper and lower limits of detection for each cytokine were based on individual analyte standard curve.

### IL-6, IL-8, and SAA1 ELISA

Centrifugation-cleared cell culture supernatants were stored at − 80°C until the day of analysis. IL-6, IL-8/CXCL8, and SAA1 concentrations were quantified using specific DuoSet® ELISA Kits (R&D Systems, DY206-05, DY208-05, and DY3019-05, respectively) according to the manufacturer’s instructions. Concentrations were normalized to the number of cells counted immediately after supernatant collection.

### Gene expression analysis by quantitative real-time PCR (qPCR)

Total RNA was extracted using the TRIzol™ Reagent (Invitrogen, #15596026), and cDNAs were obtained using the SuperScript Vilo kit (Invitrogen, #11754050) according to the manufacturer’s instructions. For the targets *CXCL1* (Hs00236937_m1), *CXCL8* (Hs00174103_m1), *SAA1* (Hs07291672_g1), and *SAA2* (Hs01667582_m1), qPCR assays were performed using TaqMan Universal PCR Master Mix (#4369016) and gene expression assays from Applied Biosystems. Gene expression was normalized using *HPRT1* (Hs02800695_m1) as housekeeping gene. For the targets *CXCL5*, *CXCL6*, and *CXCL7*, qPCR assays were performed using SsoAdvanced Universal SYBR Green Supermix (Bio-Rad Laboratories, #1725271). *GAPDH* was used as housekeeping gene. Specific primer sets were used, and primers sequences are available upon request. Reactions were performed using an AriaMx 3005 Real-Time PCR System (Agilent Technologies). Data were analyzed with the 2^−ΔΔCt^ method using the average of control samples for the ΔΔ calculation.

### Neutrophils isolation

Neutrophils were isolated from peripheral blood of healthy donors according to [47]. Briefly, pellet obtained from density gradient centrifugation as for peripheral blood mononuclear cell (PBMC) preparation containing polymorphonuclear cells were subjected to several steps of red blood cell lysis with buffer containing 0.155 M NH_4_Cl, 12 mM NaHCO_3,_ and 0.1 mM EDTA. Polymorphonuclear cells were resuspended in PBS and counted. For the analysis of viability and purity, a small amount of isolated polymorphonuclear cells (0.2 × 10^6^ cells) were stained with Zombie Violet™ Fixable Viability Dye (BioLegend, #423114) and then with FITC-conjugated anti-CD10 mAb (BD Biosciences, #347503), APC-conjugated anti-CD15 mAb (Immunotools, #21890156), and PE-conjugated anti-CD16 mAb (BD Biosciences, #332779). Samples were analyzed by flow cytometry with a CytoFLEX cytometer from Beckman Coulter, and data elaborated using FlowJo software v.10.7.1 (FlowJo, OR, USA). Gating strategy to identify neutrophils is shown in Supplementary Fig. 2.

### Neutrophil migration assay

Migration of fresh isolated neutrophils was assessed in transwells (24 wells/plate) with inserts made of 3 mm-pore membrane (Corning Costar, #3415). The lower chamber was loaded with 600 μl of attracting medium according to the experimental design, while 0.5 × 10^6^ neutrophils were loaded in the upper chamber with 100 μl. Migration assay was performed in the absence or presence of 1 μg /ml neutralizing α-human CXCL8 mAb (R&D Systems, MAB208) placed both in the upper and lower chambers. After 1 h at 37°C, the number of neutrophils migrated across the filter into the lower chamber was counted by flow cytometry with a CytoFLEX cytometer from Beckman Coulter, and data shown as percentage of migrated neutrophils in respect of loaded neutrophils.

### Neutrophil phenotyping

Cell morphology, NET formation, and ROS production were addressed after incubating 10^5^ fresh isolated neutrophils for 1 h at 37°C with conditioned media according to the experimental design. For circularity evaluation, neutrophils were fixed in 4% PFA, permeabilized with 0.2% Triton X-100, stained with AF594-conjugated phalloidin (Thermo Fisher Scientific, A12381), and counterstained with 2 μg /ml Hoechst-33342 (Thermo Fisher Scientific, H3570). Images were acquired by conventional epifluorescence microscopy using an Olympus BX51 microscope equipped with a ProgRes® MF cool monochrome camera (Jenoptik) and processed with I.A.S software ver. 009 (Delta Sistemi) for merging and pseudo-coloring adjustment. Cell circularity score was measured using the ImageJ image analysis software (Rasband, W.S., ImageJ, U. S. National Institutes of Health, Bethesda, Maryland, USA, https://imagej.nih.gov/ij/, 1997–2018) analyzing for each experimental condition 200 cells from 5 different fields acquired from two independent experiments. Briefly, images at 400 × magnification were processed performing the following actions: subtract background, adjust threshold, fill holes, analyze particles. The “overlay mask” was considered for visualizing results and the “round parameter” was considered for roundness index quantification. NETs were visualized by extracellular DNA staining with NucGreen™ Dead 488 ReadyProbes™ Reagent (SYTOX™ Green) (Invitrogen, R37109), a fluorescent membrane-impermeable DNA dye, according to the manufacturer’s instructions. Cells were counterstained with 2 μg /ml Hoechst-33342 (Thermo Fisher Scientific, H3570). When indicated (ctr +), neutrophils were stimulated with 50 ng/ml PMA (Sigma-Aldrich, P1585) for 1 h to induce NET formation. Medium alone with FBS was used for basal background (ctr-). Images were captured by fluorescence microscopy as described above. CellROX® Green Reagent from Invitrogen (C10444) was used to estimate intracellular ROS according to the manufacturer’s instructions. Neutrophils were treated with 1 μg /ml LPS (InvivoGen, TLRL-EBLPS) for 1 h as positive control. The fluorescence resulting from CellROX® Reagent oxidation was quantified by flow cytometry considering the median fluorescence intensity (MFI) and data shown as fold increase in respect to the average of proliferating samples. For the evaluation of cell debris uptake, target cells were fluorescently labeled with 1.25 μM 5(6)-Carboxyfluorescein diacetate N-succinimidyl ester (CFSE) (Sigma-Aldrich, #21888) and after plating co-cultured with neutrophils at effector:target ratio of 1:2 for 2 h at 37°C. Neutrophils were then collected and analyzed by flow cytometry.

### Statistical analysis

All statistical analyses were carried out using GraphPad Prism 8.0.2 (San Diego, CA, USA). For comparisons between two groups, a two-tailed unpaired t-test was used. For multi-group comparison, a one-way or two-way ANOVA with Tukey’s post hoc test was performed.

## Results

**Fig. 1:**
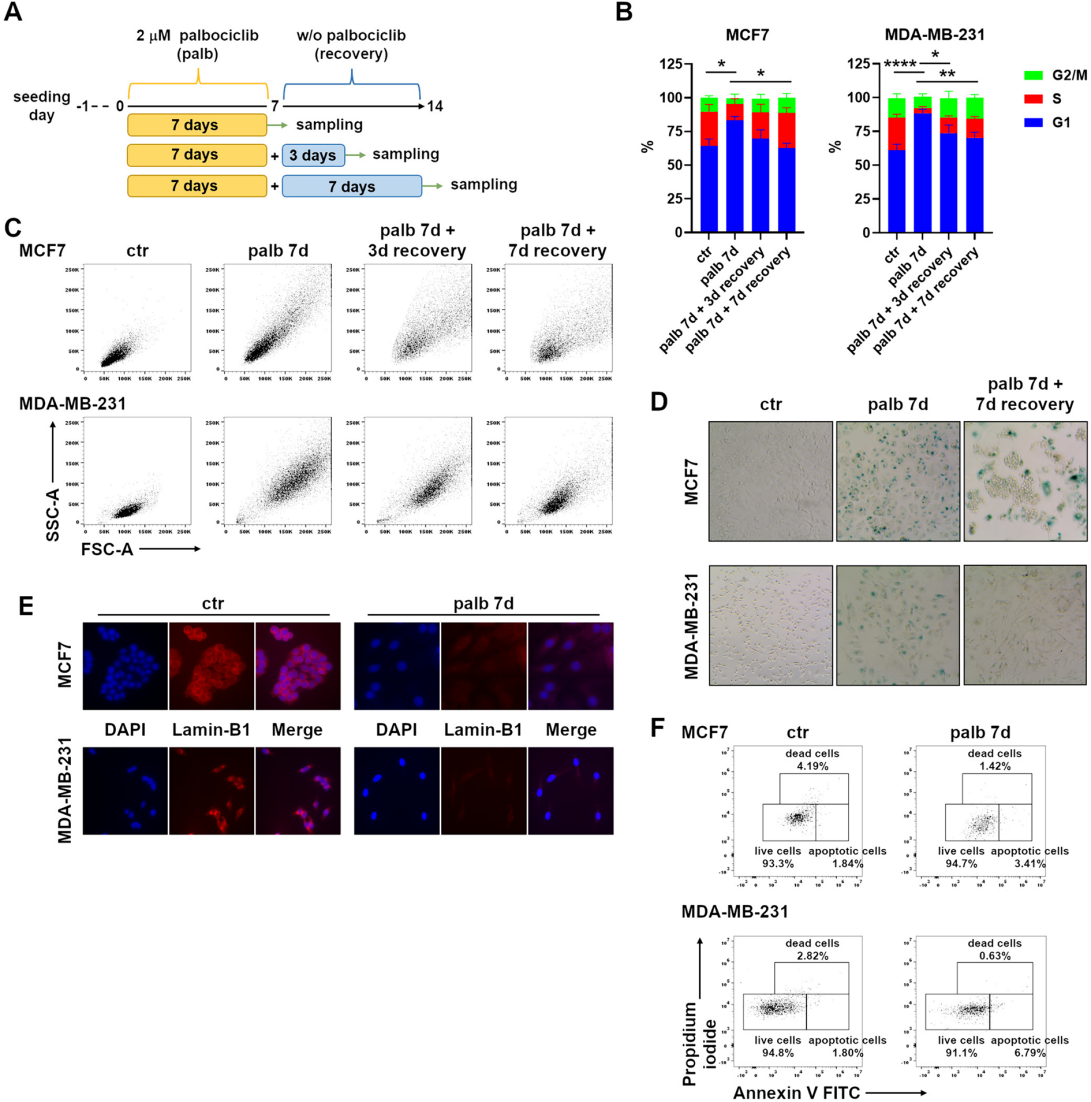
Palbociclib induces reversible senescence in breast cancer cell lines. **A** Diagram depicting the experimental design. **B** Cell cycle analysis of MCF7 and MDA-MB-231 cells treated with 2 μM palbociclib as outlined in Fig. 1A. Bars show mean ± SEM from three independent flow cytometric experiments. Statistical analysis was performed using two-way ANOVA followed by Tukey’s multiple-comparisons test. *P < 0.05, **P < 0.01, ****P < 0.0001 comparing the G1 phase. **C** MCF7 and MDA-MB-231 cell morphology after palbociclib treatment as evaluated by forward and side scatter flow cytometric parameters. **D** Senescence-associated β-galactosidase (SA-β-Gal) assay of MCF7 and MDA-MB-231 cells after one week of 2 μM palbociclib and a further week without palbociclib. Blue staining marks senescent cells. Images are representative of more than three independent experiments. Magnification 200 ×. **E** Representative immunofluorescence images of DAPI and Lamin-B1 staining of MCF7 and MDA-MB-231 cells treated with 2 μM palbociclib for 1 week. Magnification 400 ×. **F** Cell death and apoptosis of MCF7 and MDA-MB-231 cells upon palbociclib treatment as evaluated by Annexin V assay. Representative dot plots of two independent experiments

To validate our model of therapy-induced senescence (TIS), we firstly quantified the cytostatic effect of the CDK4/6 inhibitor palbociclib by analyzing the cell cycle of human breast cancer cells following palbociclib treatment. We treated both estrogen-sensitive MCF7 and metastatic triple negative breast cancer MDA-MB-231 cell lines with 2 μM palbociclib for 7 days. Cells were further analyzed after washing out the drug from the culture medium at days 10 (7 days of treatment plus 3 days of recovery) and 14 (7 days of treatment plus 7 days of recovery) to verify the reversibility of the result (Fig. 1A). Palbociclib was able to induce a reversible cell cycle arrest in the G1 phase (Fig. 1B and Supplementary Fig. 1) along with an enlargement of the cell size and increased granularity (Fig. 1C), which reverted after palbociclib removal. To ascribe the transient proliferation arrest to the establishment of a senescence program, we performed the senescence-associated β-galactosidase (SA-β-Gal) assay (Fig. 1D). As additional marker of cellular senescence, we evaluated the loss of perinuclear lamin-B1 by immunofluorescence (Fig. 1E). Besides triggering senescence, palbociclib treatment had little impact on cell death or apoptosis induction as indicated by Annexin V assay (Fig. 1F). Overall, our results show that palbociclib induces a reversible cell cycle arrest with features of cellular senescence in breast cancer cells, prompting us to further study its immunomodulatory effects.

**Fig. 2:**
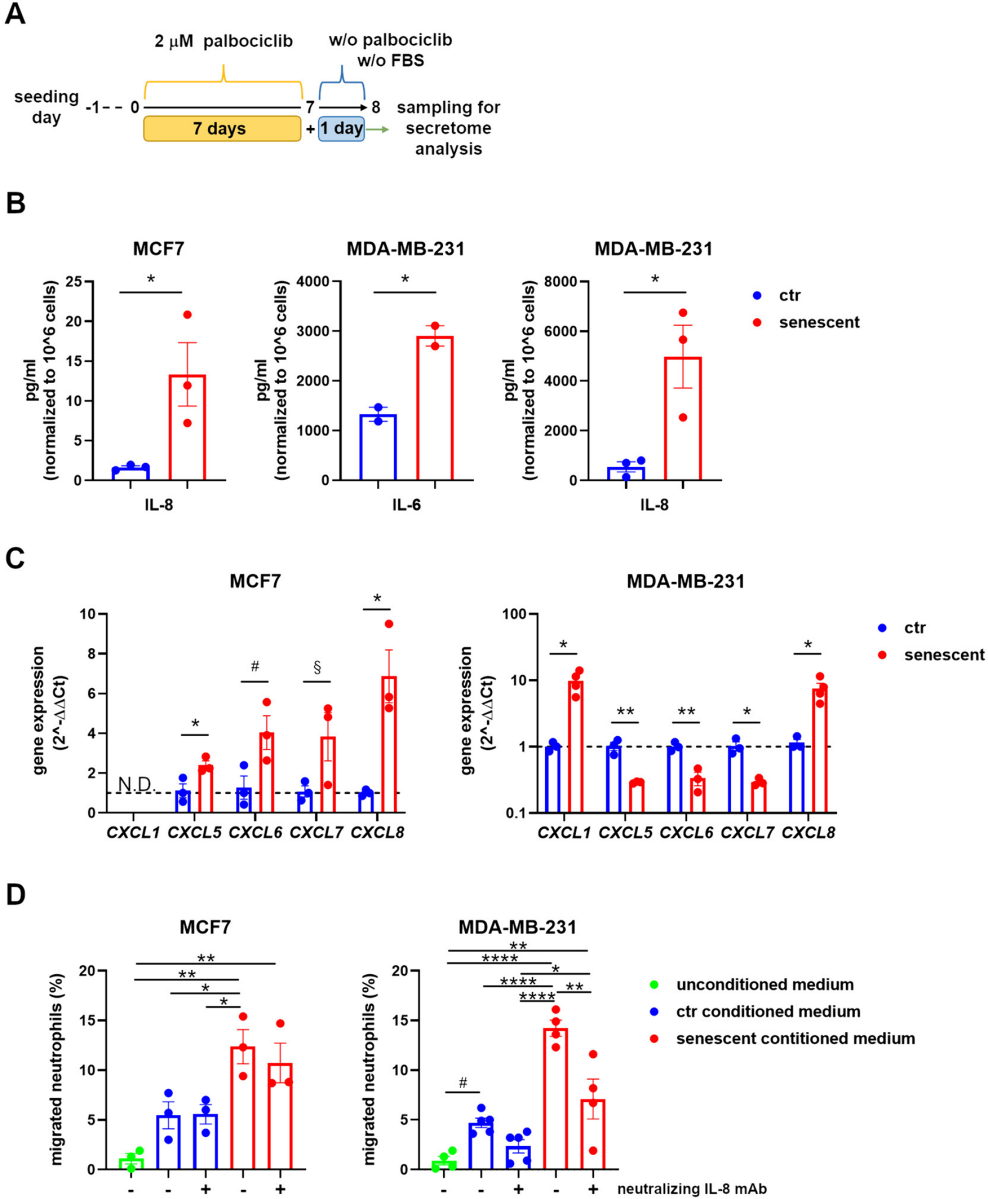
Senescent MCF7 and MDA-MB-231 cells recruit neutrophils. **A** Diagram depicting the experimental design for conditioned medium analysis. **B** Quantification by ELISA of IL-6 and IL-8 secreted by control proliferating and senescent MCF7 and MDA-MB-231 cells in 24 h-conditioned media as outlined in Fig. 2A. Bars show mean ± SEM and statistical significance was determined by unpaired two-tailed Student’s t-test. *P < 0.05. **C** Quantitative real-time PCR of the indicated CXCR2 ligands from total RNA extracted from control proliferating and senescent MCF7 and MDA-MB-231 cells. Data are reported as fold increase by using the 2^−ΔΔCt^ method and the average of control samples for the ΔΔ calculation. Bars show mean ± SEM from at least three independent experiments. Statistical analysis was performed using multiple t-test (one for each gene of interest). *P < 0.05, **P < 0.01, ^#^*P* = 0,055, ^§^*P* = 0,092. N.D. = not detected. **D** Percentage of neutrophils migrated toward conditioned media obtained from control proliferating and senescent MCF7 and MDA-MB-231 cells. Migration toward medium alone (unconditioned medium) was considered as background. Experiments were performed both in the absence and in the presence of neutralizing IL-8 mAb as indicated. Bars show mean ± SEM from at least three independent experiments. Statistical analysis was performed using one-way ANOVA followed by Tukey’s multiple-comparisons test. *P < 0.05, **P < 0.01, ****P < 0.0001, ^#^*P* = 0.087

We focused our attention on the SASP composition, which can widely shape the tumor microenvironment and act on neutrophil recruitment and activation. To this aim, we analyzed the SASP of MCF7 and MDA-MB-231 cells treated with 2 μM palbociclib for 7 days (hereafter named senescent cells) by performing a cytokine screening assay. In particular, we carried out a magnetic bead-based multiplex assay for the Luminex® platform with 24 h-conditioned media (Fig. 2A). The cytokines measured are listed in Table 1. Secretion of CCL2, CXCL10, and IL-1β was increased by senescent MCF7 and MDA-MB-231 cells compared to control proliferating cells (Table 1), confirming the inflammatory feature of a bona fide SASP. IL-6 and IL-8 (CXCL8) are two key factors of the innate immunity affecting neutrophils and frequently upregulated in the SASP, thus they were analyzed separately by ELISA. Both cytokines were largely secreted by senescent MDA-MB-231 cells, while no IL-6 and only a slight increase of IL-8 was observed in senescent MCF7 cell-derived samples (Fig. 2B). These results are in accordance with published data about the inhibitory control of the estrogen receptor over IL-6 and IL-8 expression [48–50]. The presence of IL-8 in the SASP prompted us to investigate whether neutrophils can be effectively recruited by senescent cells. To this aim, we firstly analyzed the expression of CXCL1, CXCL5, CXCL6, CXCL7, and CXCL8, ligands of the chemotactic receptor for neutrophils CXCR2, by quantitative real-time PCR (Fig. 2C). Both cell lines, even if with a different gene profile, showed upregulation of a variety of chemokines, supporting the hypothesis that senescent cells can actively recruit neutrophils. To verify this hypothesis, we evaluated the capacity of conditioned media of attracting neutrophils. Neutrophils were isolated from the blood of healthy donors and assessed in a trans-well migration assay. Neutrophil enrichment, as estimated by CD10, CD15, and CD16 staining, was above 95% and vitality above 95% (Supplementary Fig. 2). A neutralizing anti-IL-8 antibody was used to verify IL-8 specific contribution to migration. Senescent cell-conditioned media enhanced neutrophil migration compared to control proliferating cell-conditioned media. In addition, the migration induced by MDA-MB-231 cell-conditioned medium was partially reverted by IL-8 blocking, in agreement with the high expression of IL-8 measured for this cell line (Fig. 2D). These results demonstrate that tumor cells led to senescence by palbociclib promote neutrophil recruitment.

**Table 1:** SASP analysis. Cytokines from conditioned media were quantified by a multi-analyte human magnetic Luminex® assay

Cytokine (pg/ml)	MCF7		MDA-MB-231	
	Ctr	Senescent	Ctr	Senescent
**CCL2**	**76.03**	**315.85**	**36.50**	**242.09**
CCL27	< LOD	< LOD	< LOD	< LOD
CCL3	< LOD	< LOD	194.65	210.88
CCL4	188.99	205.08	304.92	304.92
CCL7	< LOD	< LOD	< LOD	< LOD
Chemerin	< LOD	< LOD	1241.74	1366.73
CX3CL1	< LOD	< LOD	< LOD	< LOD
**CXCL10**	**4.73**	**40.64**	**13.42**	**34.77**
CXCL9	998.77	998.77	1046.58	1046.58
GM-CSF	3.48	6.21	646.85	354.65
IFN-γ	< LOD	< LOD	3.66	5.98
IL-10	2.34	3.62	3.02	3.62
IL-12	< LOD	< LOD	< LOD	< LOD
IL-13	< LOD	< LOD	< LOD	< LOD
IL-15	< LOD	< LOD	< LOD	< LOD
IL-18	14.73	18.41	20.26	22.11
**IL-1α**	< LOD	< LOD	**9.22**	**40.12**
**IL-1β**	** < LOD**	**1.61**	**3.25**	**27.58**
IL-2	12.79	20.51	94.85	107.08
IL-21	< LOD	< LOD	< LOD	< LOD
IL-28A	< LOD	< LOD	< LOD	< LOD
IL-4	< LOD	< LOD	43.00	43.00
IL-5	1.14	1.14	4.02	4.02

Cytokines upregulated by senescent cells are highlighted in bold

< LOD indicates values below the limit of detection of the assay

**Fig. 3:**
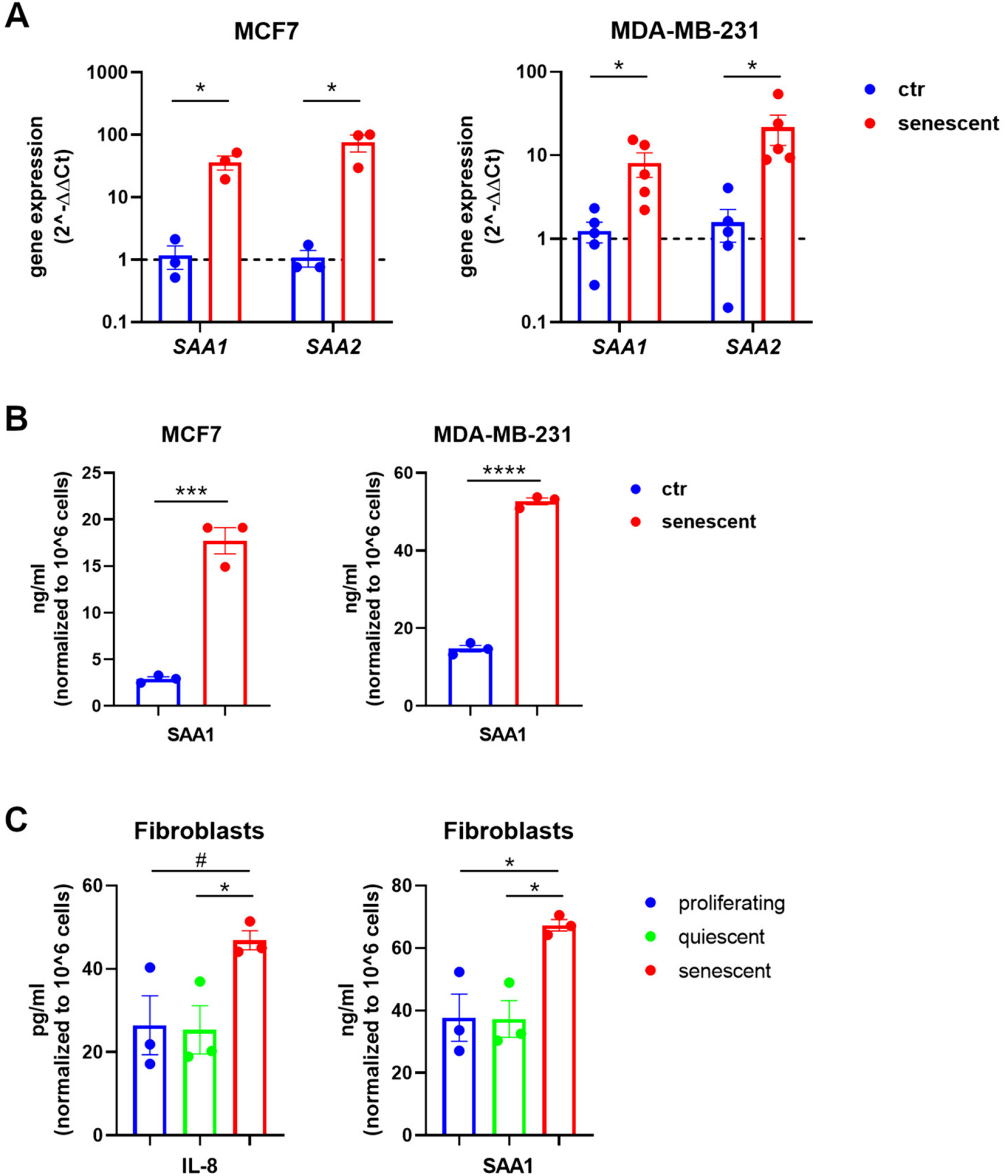
Senescent cells express recruiting and activating factors for neutrophils. **A** Quantitative real-time PCR of *SAA1* and *SAA2* genes from total RNA extracted from control proliferating and senescent MCF7 and MDA-MB-231 cells. Data are reported as fold increase by using the 2^−ΔΔCt^ method and the average of control samples for the ΔΔ calculation. Bars show mean ± SEM from at least three independent experiments. Statistical analysis was performed using multiple t-test (one for each gene of interest). *P < 0.05. **B** Quantification by ELISA of SAA1 secreted by control proliferating and senescent MCF7 and MDA-MB-231 cells in 24 h-conditioned media. Bars show mean ± SEM and statistical significance was determined by unpaired two-tailed Student’s t-test. ***P < 0.001, ****P < 0.0001. **C** Quantification by ELISA of IL-8 and SAA1 secreted by proliferating, quiescent, and senescent human foreskin fibroblasts in 24 h-conditioned media. Bars show mean ± SEM and statistical significance was determined by one-way ANOVA followed by Tukey’s multiple-comparisons test. *P < 0.05, ^#^*P* = 0.091

Besides inflammatory cytokines, the SASP includes damage-associated molecular patterns (DAMPs) [51] that are critical regulators of neutrophil activation. In particular, the acute-phase serum amyloids A1 and A2 (SAAs) have been reported to be key factors of the SASP acting in a paracrine way to reinforce the senescence phenotype [52]. Furthermore, SAA1 synergizes with chemokines in recruiting leukocytes [53]. Thus, we investigated the involvement of SAA1 as an additional chemoattractant and neutrophil-activating factor. Gene expression of *SAA1* and *SAA2* was significantly increased by senescent cells in both cell lines as evaluated by quantitative real-time PCR (Fig. 3A). We confirmed increased protein production and secretion of SAA1 by senescent cells analyzing the conditioned media by ELISA (Fig. 3B). These data suggest that neutrophils, once recruited by senescent tumor cells, may be activated by sensing DAMPs in the tumor bed. To extend the relevance of these findings and to verify that senescence per se, independently of palbociclib effects, is responsible for neutrophil engagement, we measured the amount of IL-8 and SAA1 secreted by: (1) proliferating early passage primary fibroblasts; (2) serum-starved early passage primary fibroblasts, in which the cell cycle is temporarily halted (quiescent fibroblasts); (3) late passage primary fibroblasts, which are rendered senescent by replicative exhaustion. To reach replicative senescence human foreskin fibroblasts were subcultured until they progressively acquired an enlarged, flattened morphology and stopped dividing. Cell cycle withdrawal and senescence status were verified by cell cycle analysis and SA-β-Gal assay (Supplementary Fig. 3 and Supplementary Fig. 4). We observed that only senescent fibroblasts increased IL-8 and SAA1 secretion, while quiescent fibroblasts released IL-8 and SAA1 at levels comparable to proliferating fibroblasts (Fig. 3C). These results indicate that senescent cells, regardless the type of senescence, are endowed with a program intrinsically able to engage neutrophils.

**Fig. 4:**
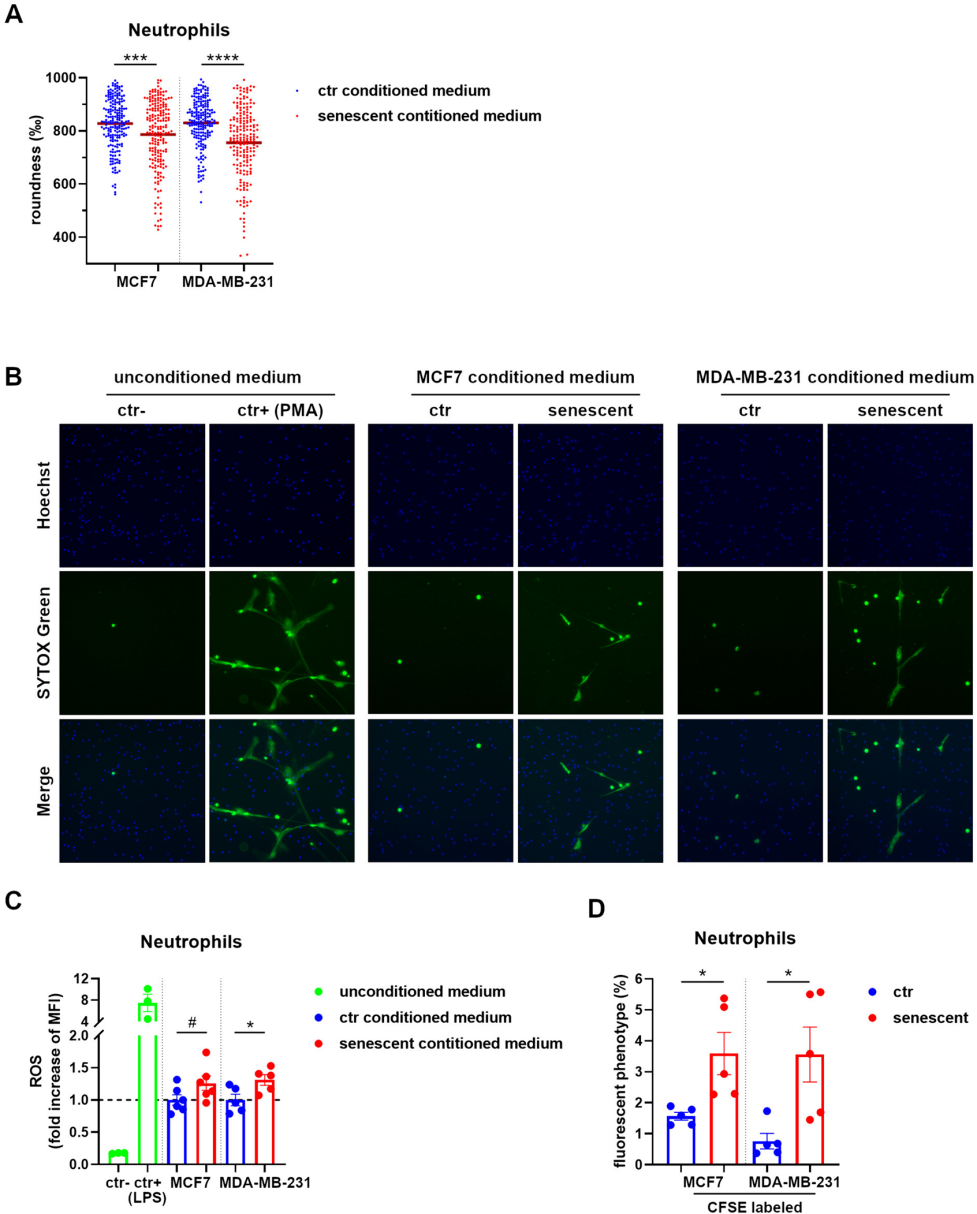
Secretome of senescent MCF7 and MDA-MB-231 cells promotes neutrophil activation. **A** Circularity score of neutrophils stimulated with the conditioned media obtained from control proliferating and senescent MCF7 and MDA-MB-231 cells. Cell shape was visualized by phalloidin staining and roundness calculated with the ImageJ software. Each dot represents the corresponding value (1000 indicates precise round shape) of a single cell. The horizontal red bar represents the mean value. Statistical analysis was performed using unpaired two-tailed Student’s t-test (one for each cell line). ***P < 0.001, ****P < 0.0001. **B** NET formation by neutrophils stimulated with the conditioned media obtained from control proliferating and senescent MCF7 and MDA-MB-231 cells. Medium alone (with FBS) and medium with PMA were used as negative and positive control, respectively. Nuclei and NETs were visualized by Hoechst and SYTOX Green staining, respectively. Images are representative of two independent experiments. Magnification 100 × . **C** ROS production by neutrophils stimulated with the conditioned media obtained from control proliferating and senescent MCF7 and MDA-MB-231 cells. Medium alone (with FBS) and medium with LPS were used as negative and positive control, respectively. ROS were quantified by flow cytometry considering the median fluorescence intensity (MFI) of ROS-specific fluorogenic probes. Data are reported as fold increase in respect to the average of corresponding  control proliferating samples. Bars show mean ± SEM from at least three independent experiments. Statistical analysis was performed using unpaired two-tailed Student’s t-test (one for each cell line). *P < 0.05, ^#^*P* = 0.088. **D** Evaluation of the uptake by neutrophils of CFSE-labeled control proliferating or senescent MCF7 and MDA-MB-231 cells as evaluated by flow cytometry. Graphs show the percentage of neutrophils acquiring the CFSE^+^ phenotype. Bars show mean ± SEM from at least three independent experiments. Statistical analysis was performed using unpaired two-tailed Student’s t-test (one for each cell line). *P < 0.05

**Fig. 5:**
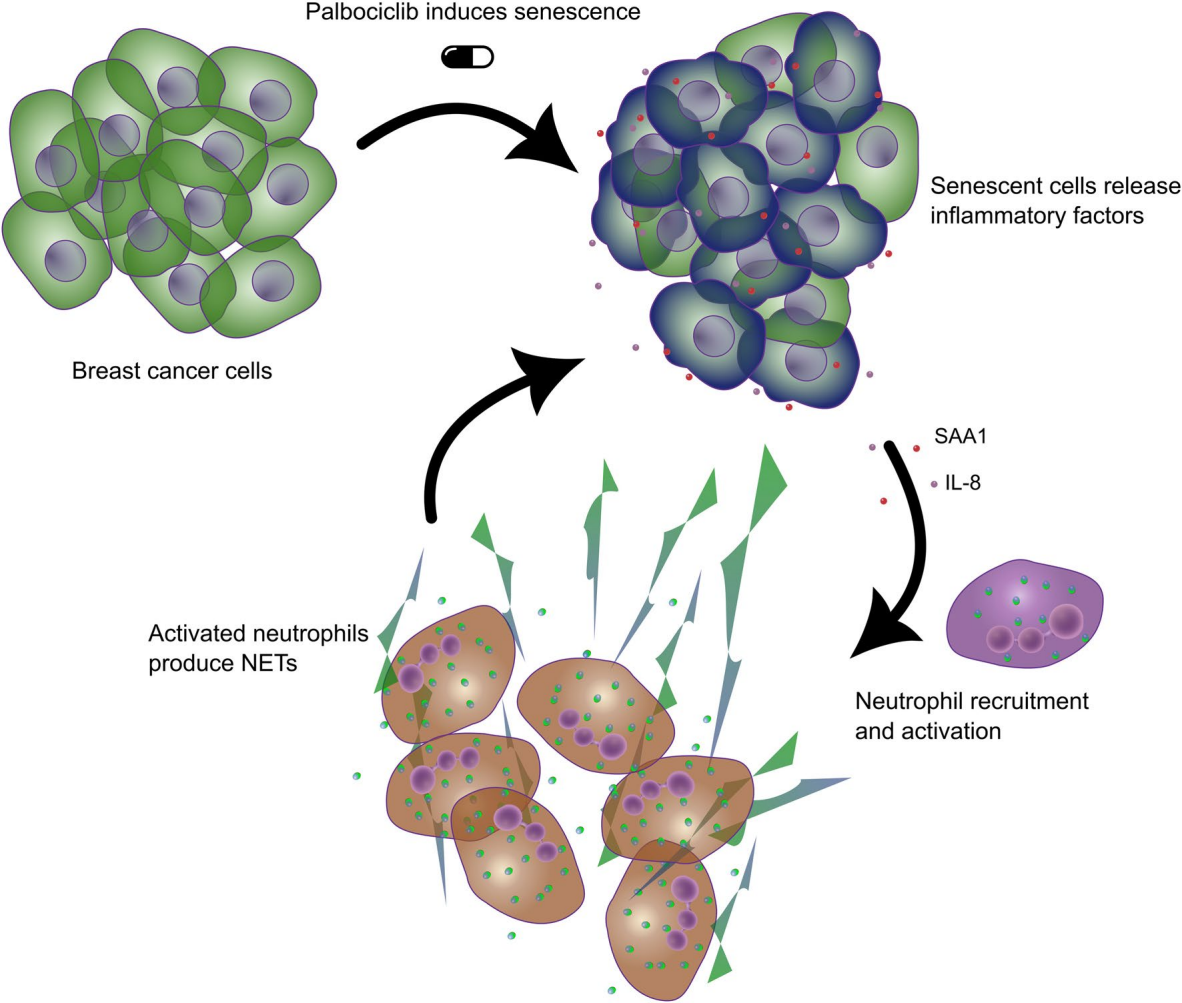
Senescence by palbociclib recruits and activates neutrophils. Breast cancer cells induced to senescence by the CDK4/6 inhibitor palbociclib release IL-8 and SAA1, which, in turn, recruit and activate neutrophils causing NET production in tumor

To assess the phenotype of neutrophils after recruitment by senescent cells, we firstly analyzed their morphology by fluorescently tagged phalloidin, as cell shape polarization is considered an early sign of neutrophil activation. Neutrophils exposed to senescent cell-conditioned media exhibited protrusions and loss of circularity, as evaluated by cell edge analysis performed by ImageJ software, while neutrophils incubated with control proliferating cell-conditioned media maintained the rounded appearance typical of resting neutrophils (Fig. 4A and Supplementary Fig. 5). The capacity of NET extrusion is a peculiar feature of activated neutrophils also in sterile inflammation. Thus, we evaluated the release of nuclear material in the form of NETs by staining neutrophils cultured with proliferating cell- or senescent cell-conditioned media with a dye labeling extracellular DNA (SYTOX Green). Strikingly, NET formation was only observed in the presence of conditioned media derived from senescent cells (Fig. 4B). NET generation is often coupled with ROS production; for this reason, we quantified ROS by using cell-permeable fluorogenic probes designed to measure ROS in live cells (CellROX® Oxidative Stress Reagents). A slight increase of ROS was detected by flow cytometry in neutrophils incubated with senescent cell-conditioned media compared to neutrophils incubated with control proliferating cell-conditioned media (Fig. 4C). Finally, to investigate a possible role of activated neutrophils in tissue remodeling, we addressed their clearance capacity of senescent tumor cells by co-culture. Neutrophils were seeded upon fluorescently CFSE-labeled proliferating or senescent MCF7 and MDA-MB-231 cells and the acquisition of the CFSE fluorescence, suggestive of cell debris uptake, was evaluated by flow cytometry. Neutrophils performed better uptake of senescent cell debris than debris of control proliferating cells (Fig. 4D and Supplementary Fig. 6), suggesting that senescent cells have increased susceptibility to neutrophil-mediated clearance. Overall, these results demonstrate that palbociclib-induced senescence, even in the absence of a permanent cell cycle arrest, is characterized by an inflammatory secretome that drives neutrophil recruitment and activation, likely triggering a local immune reaction and tissue remodeling (Fig. 5).

## Discussion

Cellular senescence has been recently proposed as an emerging hallmark of cancer [54]. However, the impact of senescent cells on tumor immune microenvironment remains incompletely understood [55]. Senescent tumor cells contribute to adjuvanticity and antigenicity but also trigger immune checkpoint expression, heterogeneously affecting innate and adaptive immunity [56, 57]. In our study, we aimed to explore the potential role of neutrophils in this context, addressing their recruitment and activation by senescent tumor cells. Given the clinical relevance of TIS in cancer treatment, we focused our study on the CDK4/6 inhibitor palbociclib, which is approved for the treatment of breast cancer and is under intense investigation for additional malignancies. In the present study, we showed that: (1) palbociclib makes tumor cells senescent with a reversible phenotype; (2) the senescent phenotype induced by palbociclib is endowed with an inflammatory secretome that can recruit and activate neutrophils through the release of different inflammatory factors, such as IL-8 and SAA1; (3) the activated neutrophils are able to perform phagocytic removal of senescent tumor cells. These findings have different implications. The reversibility of the palbociclib-induced senescence should be considered therapeutically relevant as the current clinical schedule for palbociclib is based on three weeks of administration followed by one week of stop. Long-term effects of CDK4/6 inhibition strictly depend on the duration of the treatment or genetic background (i.e., p53 status) of cancer cells as recently suggested [58, 59]. Transient cell cycle arrest may be due to an incomplete senescence program as senescence is a biological continuum and highly dynamic process rather than a terminally defined condition. Different states may exist, ranging from “light” to “deep” senescence, likely differing in some aspects. A global epigenetic reprogramming occurs during time leading to the acquisition of novel cell functions that may still revert totally or only in part [60–62]. Tumor regrowth and/or acquisition of stemness features by senescent tumor cells after cell cycle reentry have been described and must be avoided [63]. In addition, a speculative idea is now linking senescence to cancer dormancy, with yet unexplored implications [64]. It is interesting to notice that sustained activity of the mTOR pathway has been linked to the acquisition of the senescent phenotype [65, 66]. Thus, modulating the activity of the mTORC1 and mTORC2 complexes, in principle, might be exploited to avoid senescence entry or, on the contrary, to force cells into a permanent state of senescence. Alternative strategies can rely on the direct targeting of senescent cells. Indeed, senescent cells can be specifically targeted by senolytics and senomorphics, agents aimed at selectively eliminating senescent cells and abrogating/modulating the SASP, respectively [67, 68]. Steps into this direction are moving fast, and trials with senotherapeutic compounds, especially for age-related diseases, are ongoing [69]. Combined therapies implementing palbociclib with mTOR-targeting drugs or senotherapeutics could significantly improve treatments efficacy and could be accompanied by dosage lowering, with the aim of reducing side effects.

Regarding the SASP-evoked immune response, the neutrophil engagement by senescent tumor cells adds further complexity to the immune scenario. Neutrophil recruitment can be beneficial by promoting direct antitumor response in preneoplastic lesions or by switching “cold” tumors into “hot” ones. However, it may also be detrimental in the event of unresolved inflammation. In this regard, we demonstrated that activation of neutrophils upon stimulation with senescent cell secretome results in NET formation. In the context of tumors, NETosis is mostly associated with an unfavorable tumor microenvironment due to tissue damage and release of proinflammatory factors that lead to epithelial to mesenchymal transition (EMT) and angiogenesis, paving the way to metastatic processes [70]. In breast cancer, NETosis has been associated with disease worsening and vasculature adverse events [71]. Myeloperoxidase (MPO) participates in NET release by moving from the cytosol to the nucleus where it contributes to nuclear membrane breakdown. MPO is then entrapped in the extruded materials and drives the production of antineutrophil cytoplasmic antibodies (ANCAs), which have been reported to play a role in the pathogenesis of autoimmune diseases [72]. However, to which extend ANCAs or the exposure of DAMPs by NETs shape tumor immunity is largely unknown. On the one hand, ANCAs have been reported in cancer patients with vasculitis [73], thus serving as potent inflammatory factors, on the other hand, NETosis may provide adjuvant factors eliciting tumor neoantigen-driven responses. Recently, it has been shown that NETs can act as scaffold for the release of factors with antitumoral activity, i.e., cathepsin G, or, in the opposite direction, promoting EMT in cancer cells, i.e., TGF-β [74, 75].

Our secretome analysis uncovered SAA1 as a major factor of the SASP of palbociclib-induced senescent tumor cells. This acute-phase protein is a serum factor whose precise biological functions are still unresolved [53, 76, 77]. Interestingly, SAA1 (primarily through its derived peptides) appears to have differing impact on neutrophil phenotype, enhancing the inflammatory response when neutrophils reach the site of injury, but pushing toward a pro-resolving function during the resolution stage of inflammation [78]. It is tempting to speculate that senescent cells, partially by modulating SAA1, orchestrate inflammation and its resolution through a coordinate program that includes neutrophil switching from an “N1” to an “N2” phenotype. From an evolutionary perspective, cellular senescence is reasonably related to tissue healing rather than tumor suppressive mechanism [79, 80]. Following tissue injury, the induction of senescence prevents the spreading of the insult by arresting the proliferation of damaged cells and then elicits tissue remodeling by providing a plethora of bioactive factors in the microenvironment. This perspective explains the ability of senescent cells to recruit the immune system to trigger their clearance and, at the same time, to promote stemness features in the surrounding cells to enhance tissue repair. Unwantedly, this mechanism is exploited by cancer cells, favoring tumor progression. Therefore, there is an urgent need to separate tumor-inhibiting from tumor-promoting effects of senescence. IL-8 is a key chemokine involved in cancer plasticity and immune suppression [81]. In our study, we reported its upregulation by senescent cells and its effect on neutrophils. Interestingly, levels of IL-8, as well as levels of IL-6 and SSA1, were reminiscent of the baseline expression in not-senescent parental cells, supporting the finding that SASP factors are strictly related to original cell type, likely due to chromatin background, as already postulated [82]. Given that the IL-8/IL-8R axis is already a therapeutic target in different clinical studies [81], this approach could be considered to mitigate the side effects of senescence. It is now emerging that senescent cells play a prominent role during aging and aging-related disorders, including cancer, thus it is of great relevance depicting the entire immune landscape induced by cellular senescence to better tailor the upcoming therapies.

### Supplementary Information

Below is the link to the electronic supplementary material.

Supplementary file1 (JPG 205 KB)

Supplementary file2 (JPG 471 KB)

Supplementary file3 (JPG 418 KB)

Supplementary file4 (JPG 276 KB)

Supplementary file5 (JPG 305 KB)

Supplementary file6 (JPG 273 KB)

## Data Availability

The data that support the findings of this study are available from the corresponding author upon reasonable request.

## Abbreviations

ANCA: Antineutrophil cytoplasmic antibody
DAMP: Damage-associated molecular pattern
EMT: Epithelial-to-mesenchymal transition
FDA: Food and Drug Administration
IL-8: Interleukin-8
LPS: Lipopolysaccharide
mAb: Monoclonal antibody
MDSC: Myeloid-derived suppressor cell
MFI: Median fluorescence intensity
MPO: Myeloperoxidase
NET: Neutrophil extracellular trap
NK: Natural killer
PMA: Phorbol 12-myristate 13-acetate
ROS: Reactive oxygen species
SA-β-Gal: Senescence-associated β-galactosidase
SAA: Acute-phase serum amyloid A
SASP: Senescence-associated secretory phenotype
TAN: Tumor-associated neutrophil
TIS: Therapy-induced senescence
