# Supplementary figures and images for: Neutrophil-activating secretome characterizes palbociclib-induced senescence of breast cancer cells
Source: Cancer Immunol Immunother. 2024 May 2;73(6):113. doi: 10.1007/s00262-024-03695-5 (PMC11063017; doi:10.1007/s00262-024-03695-5)

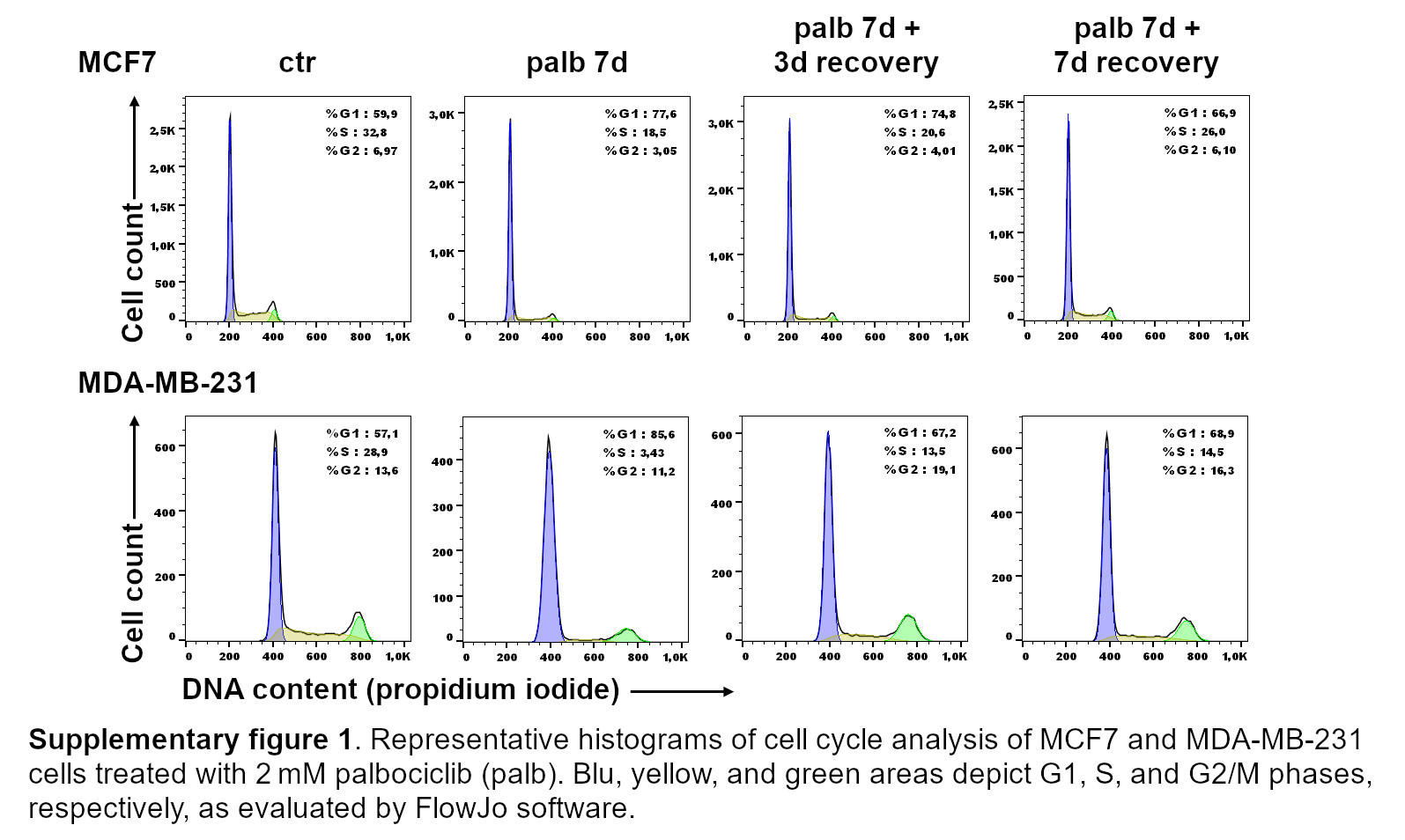

Supplement: Supplementary file 1 — Supplementary file1 (JPG 205 KB) [file 262_2024_3695_MOESM1_ESM.jpg]

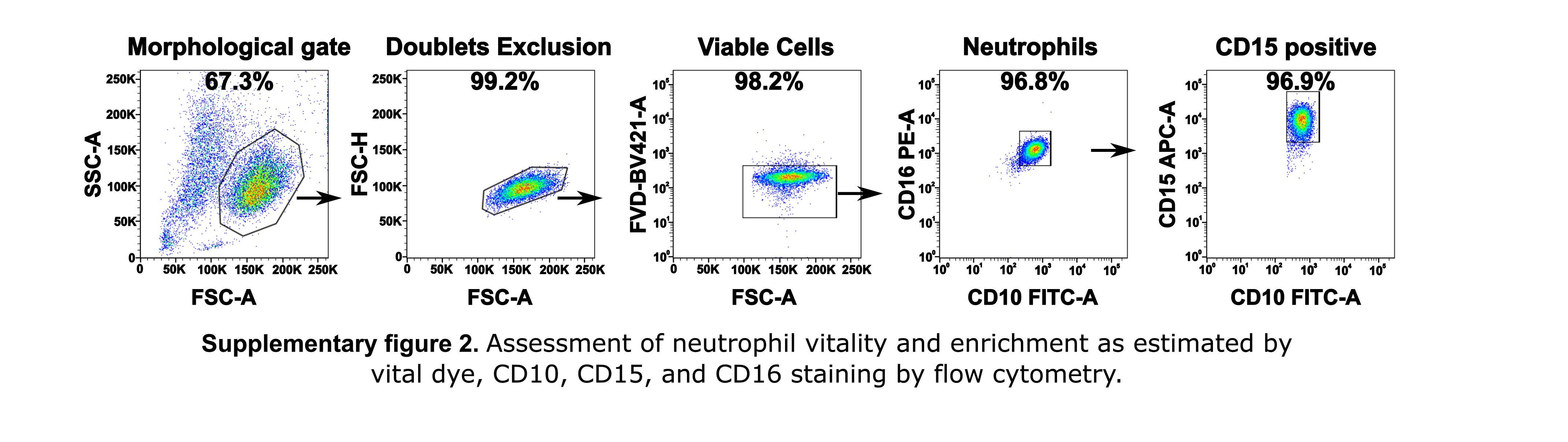

Supplement: Supplementary file 2 — Supplementary file2 (JPG 471 KB) [file 262_2024_3695_MOESM2_ESM.jpg]

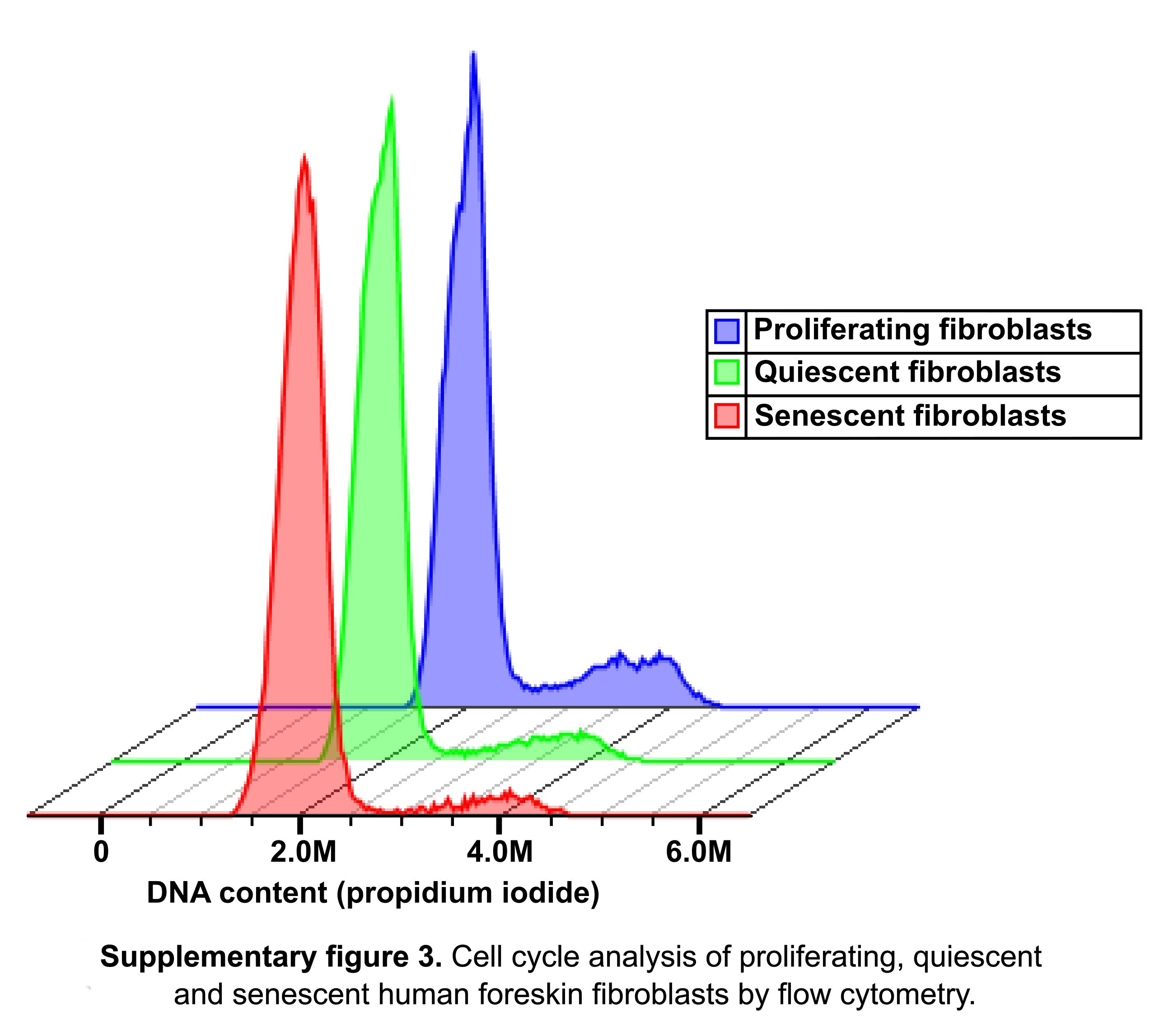

Supplement: Supplementary file 3 — Supplementary file3 (JPG 418 KB) [file 262_2024_3695_MOESM3_ESM.jpg]

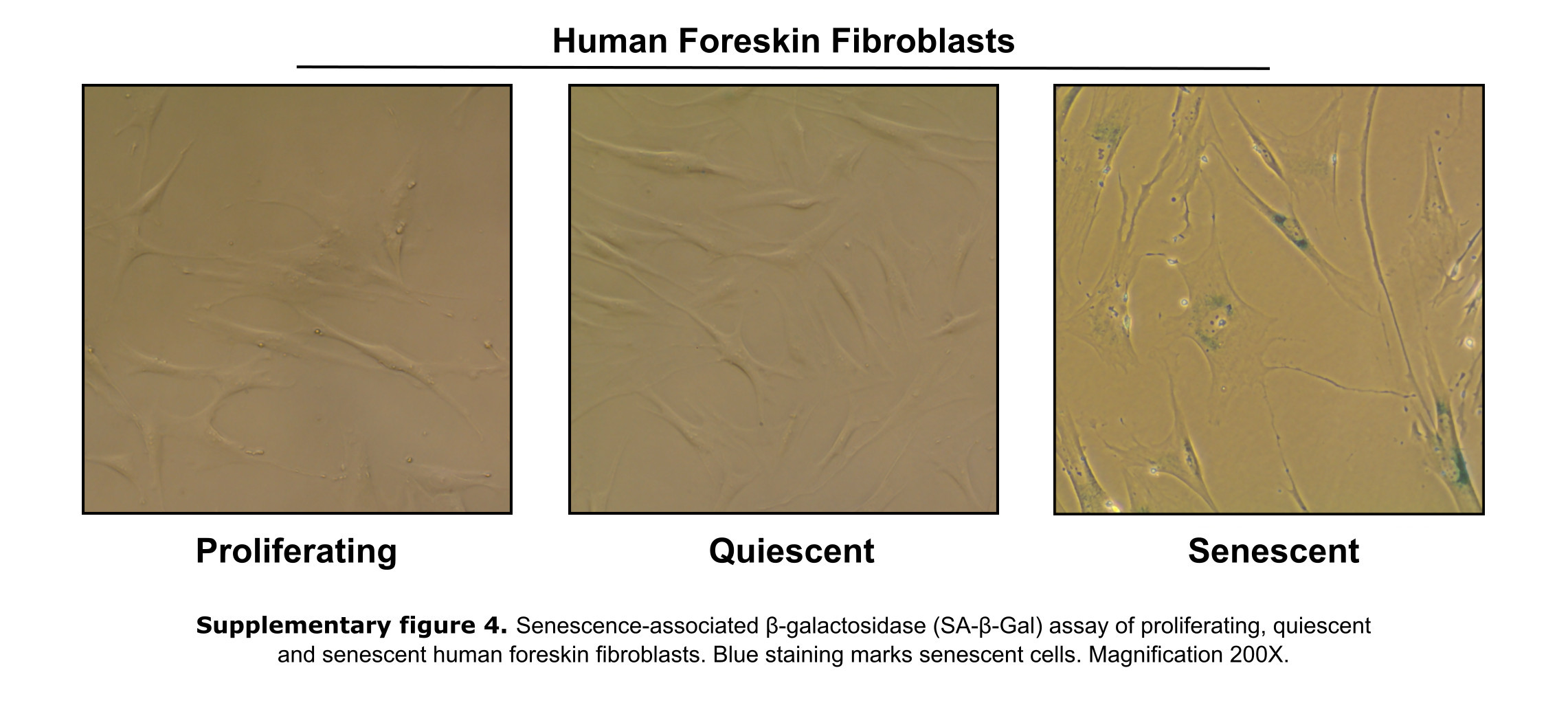

Supplement: Supplementary file 4 — Supplementary file4 (JPG 276 KB) [file 262_2024_3695_MOESM4_ESM.jpg]

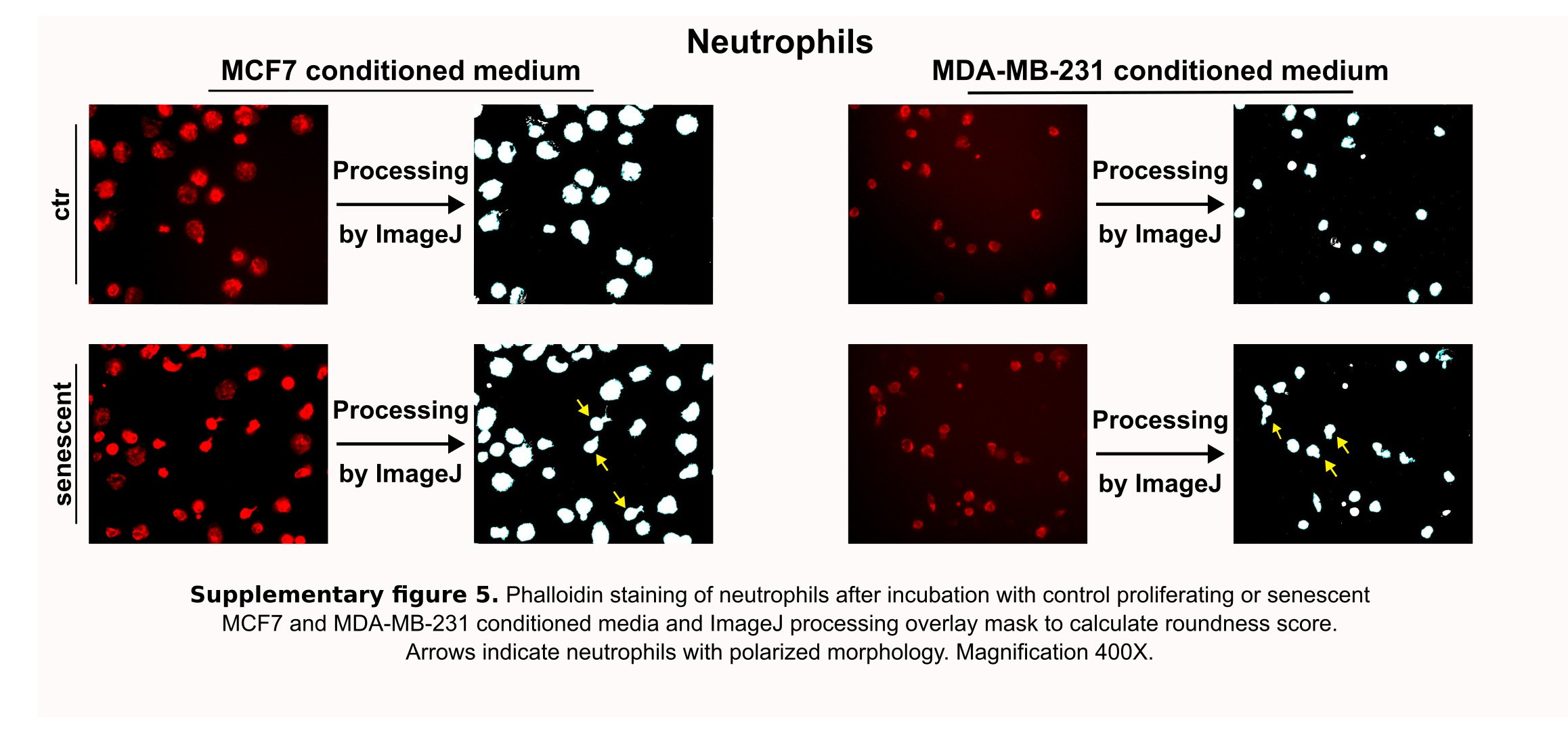

Supplement: Supplementary file 5 — Supplementary file5 (JPG 305 KB) [file 262_2024_3695_MOESM5_ESM.jpg]

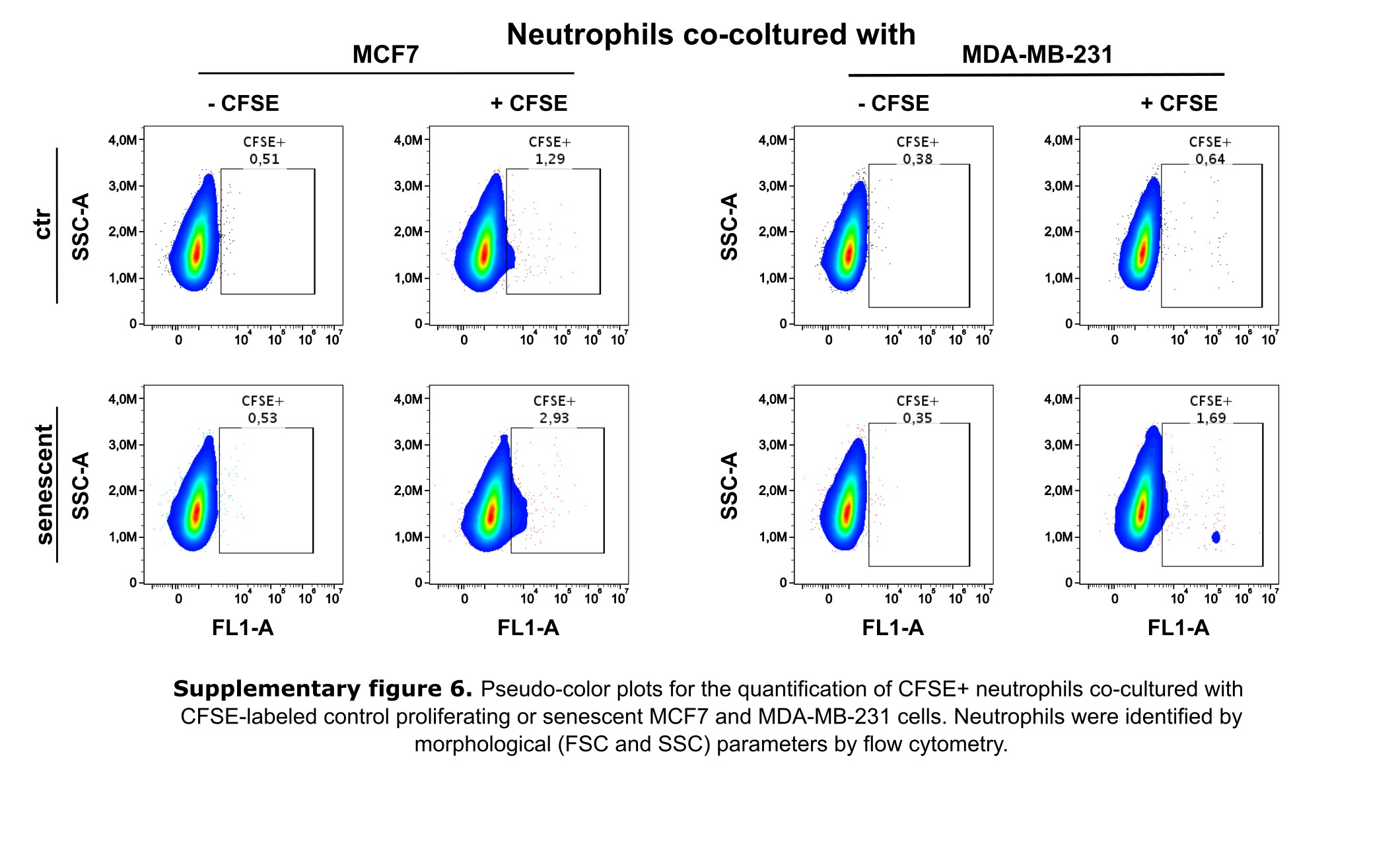

Supplement: Supplementary file 6 — Supplementary file6 (JPG 273 KB) [file 262_2024_3695_MOESM6_ESM.jpg]
